# Paired-Sample and Pathway-Anchored MLOps Framework for Robust Transcriptomic Machine Learning in Small Cohorts: Model Classification Study

**DOI:** 10.2196/80735

**Published:** 2025-10-08

**Authors:** Mahdieh Shabanian, Nima Pouladi, Liam Wilson, Mattia Prosperi, Yves A Lussier

**Affiliations:** 1 Biomedical Informatics, University of Utah, 421 Wakara Suit 140, Salt Lake City, UT, 84108, United States, 1 7736143736; 2 College of Public Health and Health Professions Dean's Office, University of Florida, Gainesville, FL, United States; 3 Center for Genomic Medicine, University of Utah, Salt Lake City, UT, United States; 4 Hunstman Cancer Cetner, University of Utah, Salt Lake City, UT, United States

**Keywords:** N-of-1, machine learning, Random Forest classifier, MLOps, weight and biases, W&B, small cohorts, ablation analysis

## Abstract

**Background:**

Approximately 90% of the 65,000 human diseases are infrequent, collectively affecting ~400 million people, substantially limiting cohort accrual. This low prevalence constrains the development of robust transcriptome-based machine learning (ML) classifiers. Standard data-driven classifiers typically require cohorts of more than 100 participants per group to achieve clinical accuracy while managing high-dimensional input (~25,000 transcripts). These requirements are infeasible for microcohorts of ~20 individuals, where overfitting becomes pervasive.

**Objective:**

To overcome these constraints, we developed a classification method that integrates three enabling strategies: (i) paired-sample transcriptome dynamics, (ii) N-of-1 pathway-based analytics, and (iii) reproducible machine learning operations (MLOps) for continuous model refinement.

**Methods:**

Unlike ML approaches relying on a single transcriptome per subject, within-subject paired-sample designs—such as pre- versus post-treatment or diseased versus adjacent-normal tissue—effectively control intraindividual variability under isogenic conditions and within-subject environmental exposures (eg, smoking history, other medications, etc), improve signal-to-noise ratios, and, when pre-processed as single- studies (N-of-1), can achieve statistical power comparable with that obtained in animal models. Pathway-level N-of-1 analytics further reduces each sample’s high-dimensional profile into ~4000 biologically interpretable features, annotated with effect sizes, dispersion, and significance. Complementary MLOp practices—automated versioning, continuous monitoring, and adaptive hyperparameter tuning—improve model reproducibility and generalization.

**Results:**

In two case studies of distinct diseases, human rhinovirus infection (HRV) versus matched healthy controls (n=16 training; n=3 test) and breast cancer tissues harboring *TP53* or *PIK3CA* mutations versus adjacent normal tissue (n=27 training; n=9 test)—this approach achieved 90% precision and recall on an unseen breast cancer test set and 92% precision with 90% recall in rhinovirus fivefold cross-validation. Incorporating paired-sample dynamics boosted precision by up to 12% and recall by 13% in breast cancer and by 5% each in HRV. MLOps workflows yielded an additional ~14.5% accuracy improvement compared to traditional pipelines. Moreover, our method identified 42 critical gene sets (pathways) for rhinovirus response and 21 for breast cancer mutation status, selected as the most important features (mean decrease impurity) of the best-performing model, with retroactive ablation of top 20 features reducing accuracy by ~25%.

**Conclusions:**

These proof-of-concept results support the utility of integrating intrasubject dynamics, “biological knowledge”-based feature reduction (pathway-level feature reduction grounded in prior biological knowledge; eg, N-of-1-pathway analytics), and reproducible MLOp workflows can overcome cohort size limitations in infrequent disease, offering a scalable, interpretable solution for high-dimensional transcriptomic classification. Future work will extend these advances across various therapeutic and small cohort designs.

## Introduction

Precision medicine seeks to personalize health care by accounting for individual differences in genetic makeup, environmental exposures, and lifestyle factors. This tailored approach becomes especially challenging when analyzing high-dimensional transcriptomic data derived from small patient cohorts (microcohorts), a scenario frequently encountered in studies of rare or infrequent diseases. Microcohorts typically involve datasets characterized by high dimensionality (approximately 25,000 transcriptomic features) juxtaposed against limited sample sizes (approximately 20 persons), conditions that commonly induce overfitting in traditional machine learning models. Advanced analytical methodologies have thus become essential in identifying robust and clinically meaningful biomarkers from these small-scale studies to facilitate personalized patient care.

A large share of the ~65,000 known human diseases are infrequent—neither rare nor common—making it difficult to assemble statistically robust cohorts without multiyear, multicenter efforts. Around 5.9% of the global population is affected by rare diseases [1], highlighting their substantial impact on global health.

Moreover, finely stratified subtypes of otherwise common diseases present similar challenges as their reduced prevalence within heterogeneous populations undermines statistical power. For example, in highly heterogeneous diseases, such as cancer, where tumor subtypes and genetic mutation profiles can vary substantially between individuals, conventional machine learning approaches often suffer from insufficient statistical power and heightened risk of overfitting. To mitigate these challenges, single-subject (N-of-1) transcriptome analytics has emerged as an innovative approach, allowing individuals to serve effectively as their own controls. By measuring within-subject transcriptomic changes and integrating these measurements into biologically interpretable pathway-level features, N-of-1 analyses significantly reduce noise and enhance the detection of biologically meaningful signals, even amidst substantial intersubject variability [2-8].

Concurrently, the emergence of machine learning operations (MLOps), inspired by DevOps practices, has significantly improved the deployment, optimization, and monitoring of machine learning (ML) models. MLOps leverage automated experiment tracking, hyperparameter tuning, and continuous integration, enhancing workflow efficiency, reliability, reproducibility, and scalability—factors essential for developing robust and maintainable models in biomedical research [9-15].

We hypothesized that integrating three complementary strategies would enhance classification accuracy and robustness in microcohort scenarios: (i) implementing MLOp frameworks to achieve robust and reproducible model performance and (ii) leveraging transcriptomic dynamics observed between paired biological samples (eg, diseased versus healthy tissues from the same individual). Paired-sample information can be incorporated in two distinct ways: (ii-a) as continuous fold-change values between matched samples or (ii-b) through single-subject (N-of-1) pathway analysis, which aggregates paired gene-level signals into biologically interpretable, ternary pathway features (upregulated, downregulated, or unchanged) across ~4000 human curated biological pathways annotated along with their respective effect sizes and significance levels.

To empirically test this hypothesis, we conducted a proof-of-concept analysis on two distinct human microcohorts, one in breast cancer (BC) (TP53 vs PIK3CA tumors) and one in human rhinovirus (HRV) infection (symptomatic vs asymptomatic), each comprising paired biological samples representing two different tissue conditions per subject. For each cohort, we systematically evaluated three distinct data transformation strategies: [i] conventional analysis using only the affected tissue per subject [ii], fold-change transformation involving the ratio of affected tissue mRNA expression to paired control tissue expression for each subject, and [iii] N-of-1-pathway transformation, summarizing individual subject-level pathway effect sizes and *P* values. The TP53–PIK3CA contrast provides a clinically relevant and mechanistically distinct testbed: both genes are frequent drivers in BC, associated with divergent transcriptomic programs and prognostic implications across the Cancer Genome Atlas and independent cohorts. Their prevalence and biological differences make them suitable paired-sample targets to evaluate whether within-subject transformations amplify signal over baseline variability.

Each of these 3 data transformations was subjected to classification modeling both with and without incorporating MLOps, resulting in a total of 12 experimental conditions across both cohorts. To further validate the robustness and relevance of features selected by the best-performing classifier, we conducted a rigorous retrospective ablation analysis. Specifically, in ablation analysis, we masked the top 20 y discriminative features from the dataset and assessed the resulting impact on classification accuracy and stability. This comprehensive analysis framework allowed us to quantify the individual contributions of key biomarkers to the model’s predictive performance.

## Methods

### Ethical Considerations

All transcriptomes were obtained as expression files from public published USA NIH datasets (gene Expression Omnibus and TCGA). Such expression data are not considered protected human information under HIPAA.

### Human Cohort Datasets

Two distinct human cohorts, spanning cancer and infection, were selected to test our framework: a BC cohort (oncogene drivers TP53 vs PIK3CA) and a HRV infection cohort (symptomatic vs asymptomatic). Both cohorts were characterized by small sample sizes, varying heterogeneity, and paired tissue samples per subject (Table 1). Processing followed published methods, ensuring prior studies' comparability [16,17].

**Table 1.** Description of the two human cohort datasets^a^.

Dataset	HRV^b^ Dataset	BC^c^ Dataset
Source-reference	GSE17156 (downloaded 9/17/2014) [18]	The Cancer Genome Atlas (TCGA-BRCA (downloaded 03/05/2019) [19,20]
Platform	Microarrays: Human Gene U133A 2.0	Illumina Hi-Seq 2000 (version 2 analyses)
Paired tissues	PBMC^d^ samples drawn before and 48 hours after HRV nasal inoculation	Primary breast carcinoma biopsies (affected) versus unaffected breast tissue margins
Experimental groups	Symptom measures before and after successful inoculation (virus present in sputum confirmed): [1] asymptomatic vs [2] symptomatic (headache, throat ache, rhinorrhea, and/or mild fever)	Somatic (tumor) mutations in either [1] *TP53* or [2] *PIK3CA* (cases with both mutations or none of these excluded)
Individuals #total	19 healthy adult volunteers 10 symptomatic for common cold 9 asymptomatic	42 patients *TP53* (23 patients) *PIK3CA* (19 patients)
Sample	38 gene expression microarray files	84 RNAseq count files

a #: count of individuals.

b HRV: human rhinovirus

c BC: breast cancer.

d PBMC: peripheral blood mononuclear cell

The classification task in BC was to identify one of two oncogene drivers that influence the treatment and prognosis, because in primary, early-stage, nonmetastatic breast carcinoma, *TP53*-mutated and *PIK3CA*-mutated tumors are generally not reliably distinguishable by histopathology alone—that is, without molecular (immunochemistry or genetic) assays. In addition, the *TP53*-driven subtype has substantially poorer 5-year survival and presents substantial resistance to therapy [21]. While the classification task in HRV was classifying symptomatic infected individuals versus asymptomatic infected ones.

Additionally, we downloaded Gene Ontology (GO) Biological Process and their gene annotations termed “gene sets,” downloaded from Ashburner et al on January 3, 2024 [22].

### Dataset Transformations

#### One Affected Tissue Transcriptome Per Individual

**Figure 1.**
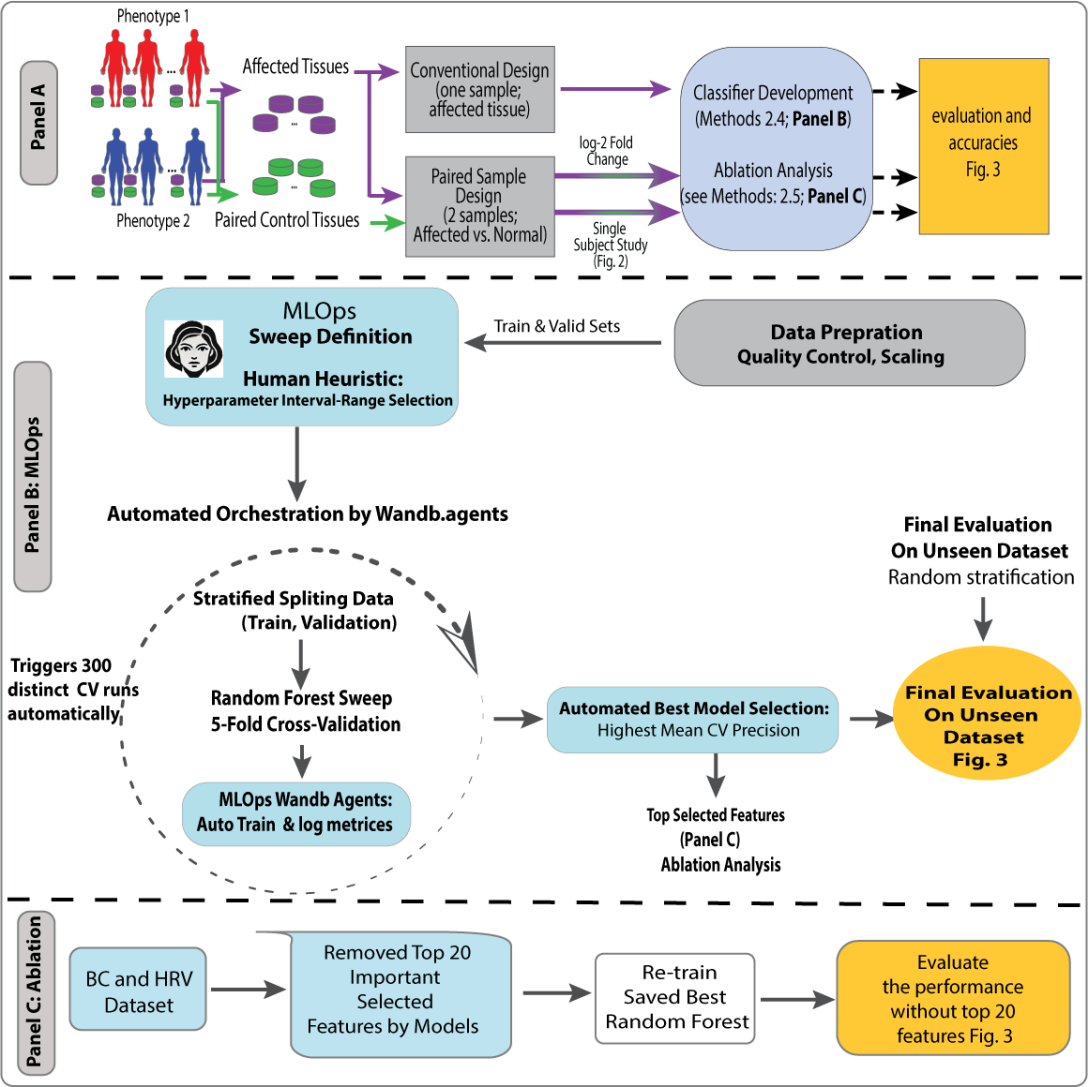
Panel A. Overview of methods and process flow of the proof-of-concept study. Classification methods are applied to 2 cohorts (Table 1), each with two distinct clinical phenotypes: (**I**) Individuals with BC, stratified by oncogenic drivers (TP53 vs PIK3CA), and (ii) HRV-infected patients (symptomatic vs asymptomatic). Each subject provides 2 samples under different conditions: (i) BC—within-subject comparison of cancerous tissue vs. unaffected margins, and (ii) HRV—within-subject comparison before versus during infection. Six classification experiments are conducted on each cohort’s extracted transcriptomes, evaluating 3 complementary classification strategies for microcohorts: (i) MLOps-driven robustness (Panel B), (ii) transcriptome dynamics between paired samples (eg, exposed vs unexposed tissue), and (iii) single-subject pathway analytics (N-of-1; details in Figure 2). Panel B. RF classifier pipeline of the BC dataset. The RF classification workflow consists of 5 key steps after extracting an unseen evaluation set: (i) hyperparameter tuning using Weights & Biases MLOp sweep definition, (ii) human-in-the-loop expert heuristics to assess failure patterns and overfitting (YAML‐based sweep configuration: criterion, max_depth, max_features, n_estimators via wandb.sweep function), (iii) iterative model refinement via 300 resampling cycles of 5-fold cross-validation (80% samples in the training set, 20% in the validation set, orchestrated by W&B MLOps (wandb.agent), (iv) MLOps Automated Best Model Selection, and (v) final evaluation on unseen dataset. Panel C. Retroactive feature ablation analysis: feature importance is assessed in both datasets to evaluate the impact of individual features on classification performance. BC: breast cancer; HRV: human rhinovirus; RF: Random Forest;

Most conventional transcriptome classifiers typically analyze a single transcriptome derived from the affected tissue of each individual. To evaluate the accuracy achievable with traditional classification methods using one sample per individual, we used the affected tissue of the datasets and did not use the paired control tissue. The BC cohort [16]included 22,279 TMM (trimmed mean of M values) normalized gene expression [23] values from 42 individuals, and two samples per individual (BC and unaffected margins). The HRV cohort [16]included 20,502 RMA-normalized Affymetrix GeneChip expressions of probe sets from 19 individuals and two samples per individual (peripheral blood mononuclear cells 48 h before HRV inoculation and after successful inoculation and shedding of virus) (Figure 1, Panel A).

**Figure 2.**
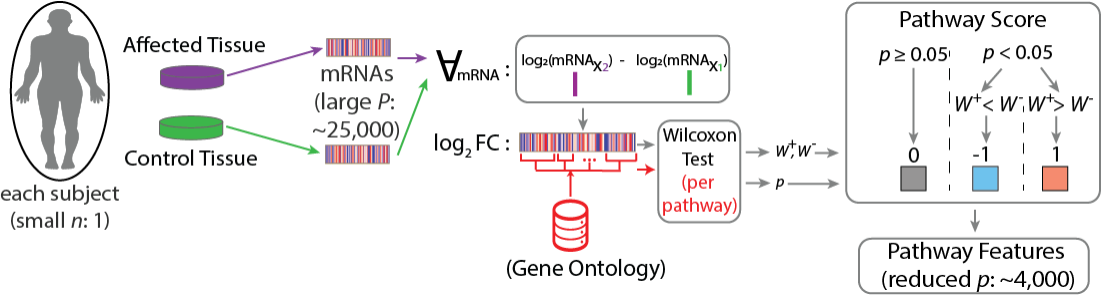
Description of the N-of-1-pathway Wilcoxon analytics in each single subject. We used the “N-of-1-pathways” method [17], which aggregates paired RNA-level signals of each subject into pathway-level effect sizes, conducts a nonparametric Wilcoxon test comparing the pathway-associated mRNAs in each Gene Ontology (GO) Biological Processes [*P* <.05; other thresholds studied elsewhere [2,3,6,16,17] for each subject, enabling downstream classification over a smaller number of human-interpretable GO features. This method identifies significantly altered mRNA sets associated with a pathway between two samples of one subject, yielding 4,442 GO mRNA sets in the BC cohort and 2,332 GO mRNA sets in the HRV cohort. The output consists of ternary matrices indicating response status 1−: negatively regulated, +1: positively regulated, and 0: unaltered GO pathway. For each GO pathway, we compute FC of mRNA expression values between the affected and control tissue of a single individual. A Wilcoxon test is then performed on these values, where the sum of positive ranks (W+) and negative ranks (W−) determine the test statistic W by min⁡(W+,W−). The relative magnitude of W+versus W− indicates whether the pathway is positively or negatively regulated in a significant test (eg, W+>W− indicates a positively regulated pathway; W−>W+ indicates a negatively regulated pathway, and a nonsignificant test indicates an unaltered pathway). HRV scores were refined with a coefficient of variation <31%. FC: fold change; n: number of subjects; *P* or *p*: number of features (transcripts); W+: statistically significant Wilcoxon test with up-regulated gene set (pathway score positive); W-l: statistically significant Wilcoxon test with downregulated gene set (pathway score positive); negative): mRNA=messenger RNA; X2: indices of the affected tissue; X1: indices of the control tissue.

#### Paired Samples: One Affected Tissue Transcriptome and One Control Tissue Per Subject.

We calculated the fold change by dividing the expression of each mRNA value of the affected tissue by that of the control tissue, in each subject, in each dataset, followed by a log_2_ transformation [6]. Single-subject studies (N-of-1-pathways) are described in Figure 1 Panel A and Figure 2.

### Model Selection

We evaluated several ML models, including Random Forest (RF), XGBoost, Support Vector Machine (SVM), and Logistic Regression. Random Forest was ultimately chosen due to its robustness, capacity to model nonlinear interactions, and superior predictive performance in identifying symptomatic patients and relevant gene sets. Multimedia Appendix 1 provides a comparative analysis of the ML models, highlighting the factors underlying RF’s superior performance.

### Classification, Cross-validation, and MLOps

Model robustness was evaluated in both datasets using 5-fold cross-validation. The RF model was integrated into the Weights & Biases (W&B) MLOp framework (W&B v0.17.0, Python 3.11.4) [24] to systematically identify features whose interactions significantly contribute to class differentiation. MLOps facilitated robust experiment tracking, hyperparameter optimization, and model monitoring, applying consistent hyperparameter ranges across the BC dataset (42 samples) and the HRV dataset (19 samples). This setup allowed us to assess MLOps’ effectiveness in guiding hyperparameter tuning and model tracking while maintaining human oversight. This study was designed to compare the ability of different combinations of data transformations (single-sample per individual, FC, N-of-1-pathways analytics) to improve performance in small human cohorts (small n<30 individuals) with high feature dimensionality (very large *p*, transcriptomes=25,000 mRNA features)

In W&B MLOps, the *sweep.yaml* file configured hyperparameter sweeps by defining key parameters, search strategies, optimization metrics, and other relevant settings for systematic model optimization. Python’s *StratifiedKFold* strategy ensured class proportion consistency and class imbalance across 5 folds, and this process was repeated across 5 iterations with different folds serving as the validation set, constituting a stratified 5-fold cross-validation unbiased model selection protocol. Accuracy, precision, and recall performance metrics were calculated across cross-validation folds and held-out unseen test sets (Tables2  3). The held-out unseen test partition was sequestered throughout model development and accessed only once, after cross-validation and hyperparameter selection were completed, ensuring that no tuning decisions were informed by test data. To refine hyperparameter ranges, a human expert in the sweep configuration loop revised the best hyperparameter intervals using the *sweep.yaml* configuration. This YAML file specifies the parameters to be tuned, the search strategy, optimization metrics, and other pertinent settings (Figure 1, Panel B). To further evaluate generalizability given the limited cohort sizes, we performed a learning curve analysis and accompanying power calculations; results are provided in Multimedia Appendix 2, which details experimental reproducibility safeguards (eg, immutable YAML configurations, dataset/hyperparameter hashes, deterministic folds, and logging of all trials to MLOps). Methods for tracing RF classifier decisions to biological mechanisms are addressed in Multimedia Appendix 3.

**Table 2.** Performance summary of analysis in human rhinovirus (symptomatic vs asymptomatic) Random Forest classifier.^a^

Feature (transcript) transformation design	Single-sample mRNAs	Two-sample (one sample in each condition) mRNAs	
		Fold change	N-of-1 pathways (single-subject studies)
Number of features and samples			
Number of mRNA transcripts	22,279 features	12,496 features	553 (no. of GOs^b^)
Training samples	15	15	16
Validation samples	4	4	3
Cross validation (CV) values			
CV accuracy: mean (SD)	.85 (.16)	.95 (.15)	.88 (.14)
CV precision: mean (SD)	.87 (.22)	.97 (.21)	.92 (.14)
CV recall: mean (SD)	.85 (.16)	.95 (.17)	.90 (.16)
CV F1: mean	.86	.91	.96
Selected feature count	266 mRNAs	112 mRNAs	42 GOs
Hyperparameters			
Entropy criterion maximum depth	87	18	42
Maximum features	log2 n-estimators: 148	null n-estimators: 56	sqrt n-estimators: 24

a Fold-change model achieves highest CV precision (0.97), while N-of-1 pathway model offers greater stability with the lowest CV (SD 0.14), outperforming single-sample designs across all metrics. Corresponding 90% CIs are provided in Supplement File 5 in Multimedia Appendix 4.

b GO: Gene ontology Biological Process gene set.

**Table 3.** Performance summary analysis in the breast cancer Random Forest (*PTP53 vs PIK3CA*) classifier^a^.

Feature (transcript) transformation design	Single mRNAs	Two sample (one in each condition) mRNAs	
		Fold change	N-of-1 pathways (single-subject studies)
Number of mRNA transcripts	20,502 features	16,384 features	4442 features (no. of GOs^b^)
Training sample	27	27	27
Validation sample	6	6	6
Test samples	9	9	9
Cross validation (CV) accuracy: mean (SD)	.72 (.18)	.62 (.15)	.73 (.07)
Unseen test set accuracy			
Test accuracy	.78	.78	.89
Test precision	.78	.86	.90
Test recall	.77	.78	.90
Test F1	.78	.82	.90
Selected feature count	105 mRNAs	97 mRNAs	21 GOs
Entropy criterion maximum depth	115	148	165
Maximum features	sqrt n-estimators: 17	log2 n-estimators: 23	null n-estimators: 8

a On the unseen test set, pathway-level features achieved 12% higher accuracy and greater stability compared with fold-change and single-sample classifications. Corresponding 90% CIs are provided in Multimedia Appendix 4.

### Feature Importance, Stability, and Top-K Retroactive Feature Ablation

For each dataset and representation (single-sample mRNA, fold-change mRNA, and N-of-1 pathway scores), we trained RF under repeated, stratified 5-fold cross-validation. Within each fit, “feature importance” was computed as a mean decrease in impurity (MDI)—the sample-weighted reduction in node impurity attributable to a feature—and then aggregated across trees, folds, and repeats to yield a global ranking [25]. To assess the “stability” of per-repeat rankings, we computed (i) Spearman rank correlation (ρ) on the full ordering and (ii) Jaccard overlap of the Top-k feature sets [26-28]. *Top-K* denotes the k highest-ranked features by aggregated MDI computed on the full, unpruned feature space.

We conducted a retroactive feature ablation analysis on both datasets to assess the impact of the top-ranked features identified by our selected classifiers. To harmonize ablations across representations, we prespecified k=20 (two final models selected 21 features, motivating a common k). For retroactive ablation, we removed the top 20 features from the training, validation, and held-out test partitions. We then refit from scratch the previously selected model configuration with its exact, prechosen hyperparameters, without additional tuning or human-in-the-loop changes. The held-out test set, transformed once by dropping the same training-derived top 20 indices, was evaluated a single time. This remove-and-refit procedure estimates the marginal contribution of top-ranked features while minimizing information leakage [29]. This retraining step was conducted to measure the influence of the ablated features on performance metrics such as precision and recall (Figure 1, Panel C). Together, MDI rankings, stability metrics, and ablation results provide post hoc explainability of the model’s global feature contributions [30].

## Results

**Figure 3.**
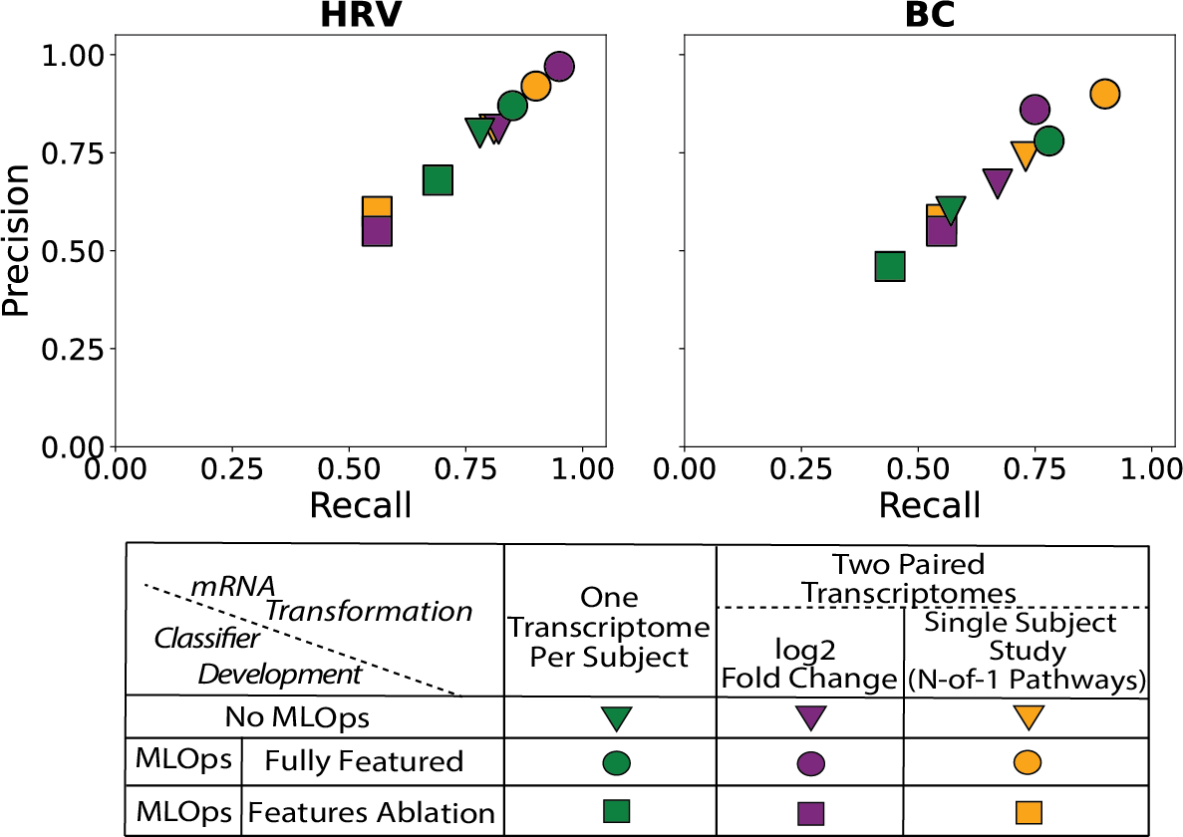
Paired sample per subject machine learning designs outperform single-sample designs. As illustrated, paired-sample per subject designs, either using log₂ fold change (purple) or single-subject N-of-1-pathway analysis (yellow), achieved higher classification accuracies compared to single-sample per subject designs (green), across both Breast Cancer (BC) and Human Rhinovirus (HRV) microcohorts. However, pathway-based classification surpassed fold-change performance in only one dataset, suggesting that the underlying biology (eg, structure of the information model) of a condition may determine whether pathway-level or gene-level (fold-change) features are more informative for classification. No MLOps indicates a conventional cross-validation run without iterative retraining or sweep-based refinement, serving as the baseline against which the orchestrated MLOp pipeline was compared. Incorporating MLOps (circles and squares) yielded an average accuracy improvement of ~14.5% compared to traditional approaches without iterative retraining (ie, single cross-validation runs). By contrast, classifiers subjected to retroactive top 20 feature ablation (indicated by squares) experienced a performance drop of approximately 25%. MLOps: Machine Learning Operations; mRNA: messenger RNA; Log2: logarithm base 2

In both datasets, RF model robustness was evaluated using 5-fold cross-validation (Methods 2.3‐2.4, Figure 1 Panels A-B). For the 42 individuals BC dataset (23 *TP53* and 19 *PIK3CA*), 80% (27 individuals) was used for training, while the remaining 20% was split into 6 individuals for validation and 9 individuals for testing, ensuring consistent evaluation. Similarly, in the HRV dataset, consisting of 19 individuals (10 symptomatic and 9 asymptomatic), the data were split into 80% (16 individuals) for training and 20% (3 individuals) for validation. The *StratifiedKFold* approach from the *scikit-learn* Python package was used to maintain consistent class proportions across folds, ensuring validation consistency and reproducibility, and preserve class proportions in every training/validation split (class imbalance results not shown). In MLOp-guided studies (Methods 2.4, Figure 1 Panel B), after testing various hyperparameter interval ranges, a human-in-the-loop (expert) confirmed the following optimal RF hyperparameters: criterion (*gini* or *entropy*), number of estimators (5 to 150), maximum features (sqrt, log2, or None), and tree depth (5 to 200). SVM and XGBoost hyperparameters are not shown as they yielded lower accuracies. As summarized in Table 2 (HRV), Table 3 (BC), and Figure 3, paired-sample–based feature transformation strategies outperformed single-sample approaches across all major evaluation metrics. In the HRV cohort, the fold-change model yielded the highest cross-validation (CV) precision (0.97±0.21 SD) and recall (0.95±0.17 SD), while the N-of-1 pathway-based classifier demonstrated superior stability, achieving lower CV SDs across all metrics, including CV precision. By contrast, the single-sample model achieved a CV precision of only 0.87±0.22 SD. To further assess generalizability given the limited cohort sizes, we performed a learning-curve analysis using N-of-1 pathways as an exemplar; these results are provided in Multimedia Appendix 2. To trace classifier-selected features back to underlying biological processes, we performed heatmap clustering of features and pathway enrichment of transcripts, provided in Multimedia Appendix 3. To address the concern that the superior performance of the N-of-1 pathway method may reflect dimensionality reduction rather than pathway biology, we performed an additional analysis applying comparable feature reduction (~4000 features) to the single-sample and fold-change models; these results are provided in Multimedia Appendix 5.

In the BC cohort, the N-of-1 pathway–based model achieved the highest test precision and recall of 0.90, reflecting an approximate 12% absolute improvement over the single-sample classifier (0.78 precision, 0.77 recall). This model also demonstrated greater stability, with a cross-validation SD approximately half that of the single-sample and fold-change approaches. These findings are further illustrated in Figure 3, which compares performance across transformation strategies. Collectively, the results demonstrate the effectiveness of paired-sample transformations—particularly when combined with MLOp-guided optimization—in improving classification accuracy and model stability in micro-cohort settings.

Retroactive feature ablation studies were conducted in breast cancer and HRV datasets (Figure 3; Multimedia Appendix 5). To assess the impact of top-ranked features on model performance, an ablation study was performed by sequentially removing the 20 highest-ranked features identified by the classifiers and retraining the optimal Random Forest model with previously tuned hyperparameters. It consisted of masking these features from the data input and retraining (Methods 2.5; Figure 1 Panel C). This analysis quantified the contribution of these features by evaluating changes in precision and recall, revealing a significant decline in predictive accuracy upon their removal. The results underscore the robustness of the selected features derived through the MLOps-driven pipeline, with performance degradation observed across all feature sets. Of note, most classifiers retained on the order of ~100 features, whereas the final BC model retained only 21 N-of-1 pathway features; nonetheless, the ablation step uniformly removed the top 20 features across all methods to maintain consistency, regardless of the total feature count. In addition, we evaluated models trained using only the top 20 features, which performed substantially better than the ablated models but below the full models (Multimedia Appendix 5), thereby quantifying both the predictive value and the limitations of this small feature subset.

## Discussion

### Principal Findings and Comparison With Previous Works

Transcriptome classifiers traditionally analyze a single transcriptome per subject, providing a baseline for evaluating the performance of standard classification methods. In our study, this conventional approach was represented by the single-sample per subject design. Specifically, the BC cohort [11] included 22,279 gene expression values normalized using the trimmed mean of M values (TMM) method [20] from 42 individuals, while the HRV cohort [6] comprised 20,502 Affymetrix GeneChip probe-set expressions normalized using Robust Multiarray Average (RMA) from 19 individuals.

We systematically compared 3 mRNA feature transformation strategies—single-sample, log₂ fold-change (paired design), and N-of-1 pathways (paired design)—across both datasets, using identical hyperparameter sweeps implemented within the W&B MLOp platform (*wandb* v0.17.0, Python 3.11.4). Among the evaluated classifiers (Random Forest, XGBoost, SVM, Logistic Regression), RF was selected for final implementation based on its ability to model nonlinear interactions and superior predictive performance in distinguishing symptomatic individuals and uncovering relevant gene sets (data not shown).

Results consistently demonstrated that paired-sample per designs outperformed single-sample designs, with up to 12% higher precision accuracy observed for the N-of-1 pathway–based approach in BC and 5% in HRV, while recall was increased by 13% and 5%, respectively. However, this performance advantage varied across datasets: while pathway-based classification outperformed fold-change in the BC cohort, fold-change achieved 10% increase in both precision and recall in the HRV dataset. The impact of pathway-level features on classification outcomes is demonstrated by their high importance rankings and the sharp ~25% accuracy drop observed in retroactive ablation, showing that the model not only learns from these features but also relies on them as key decision boundaries. Thus, the consistent finding is that 2-sample transformations outperform single-sample designs, although which representation (fold-change vs pathway) is optimal appears task- and biology-specific. At present, methods to prospectively identify which 2-sample representation will perform best in a given dataset remain undeveloped; however, the differential results here are consistent with biological granularity as oncogene-level classification in breast cancer is inherently pathway mediated, while HRV organism-level symptom classification reflects broader organismal phenotypes. These differences suggest that the underlying disease biology influences whether gene-level or pathway-level features are more informative. In our framework, pathway-level features contribute to classification by encoding coordinated transcriptomic changes into ternary indicators of pathway activation (upregulated, downregulated, or unchanged). Unlike raw expression values or continuous fold-change variables, these ternary ordinal features emphasize significant, coordinated shifts at the pathway level, providing interpretable signals that capture biological mechanisms rather than gene-level noise. These ternary variables act as global indicators of pathway perturbation, enabling the classifier to learn patterns of coordinated biological dysregulation that are not captured by individual transcripts alone. This representation reduces dimensionality by several orders of magnitude, mitigates noise from gene-level variability, and provides features with direct biological meaning.

Distinguishing TP53- from PIK3CA-driven breast cancers is clinically important: TP53 mutations predominate in estrogen receptor–negative tumors and portend poor prognosis, whereas PIK3CA mutations are frequent in estrogen receptor–positive tumors and guide PI3K/mTOR–targeted therapy [21]. Transcriptome-based classifiers that stratify TP53 versus PIK3CA mutations therefore have direct translational value for prognosis and treatment selection. Classifying transcriptome-level signals (~10⁻⁸ m) by oncogenic driver mutations—molecular alterations occurring at the nanometer scale is inherently a proximal task in the biological hierarchy, especially when contrasted with symptom-based classifications for HRV infection, which manifest at the meter scale. Moreover, early-stage primary breast carcinoma remains fundamentally a disease of genetic, genomic, and subcellular pathways. It is therefore more amenable to gene set–based transformations as conventional histology alone cannot reliably distinguish its molecular subtypes without adjunct immunohistochemical or genomic markers. In summary, paired-sample designs consistently improved precision and recall, as hypothesized; however, the optimal transformation method may vary by disease context, with some conditions favoring fold-change models and others better suited to single-subject gene set analysis.

Integrating MLOps into the modeling pipeline led to a ~14.5% improvement in classification accuracy compared with non-MLOps workflows that relied on a single cross-validation run without iterative retraining. This finding underscores the benefit of programmatic, reproducible, and feedback-driven model development. Moreover, our retroactive top feature ablation analysis, which involved retraining classifiers after removing the top 20 features, revealed a ~25% reduction in accuracy, demonstrating the importance of retaining high-contribution features in high-dimensional settings.

In the HRV microcohort, the MLOp-guided fold-change model achieved excellent precision (0.97) and recall (0.95), while single-sample designs were more susceptible to overfitting and noise due to higher dimensionality. By contrast, the N-of-1-pathway approach proved more effective in the BC cohort, which is characterized by heterogeneous tumor biology; this model achieved test precision and recall of 0.90. Conversely, MLOp-guided fold-change analysis in BC yielded lower precision (0.86) and recall (0.75), highlighting the relative strength of pathway-informed features for modeling complex biological variation.

Collectively, these results highlight how paired-sample designs—particularly when paired with MLOps—yield more accurate and interpretable models, especially in small cohort scenarios. Furthermore, our study demonstrates how expert-guided decisions about feature transformations (eg, fold-change vs pathways), integrated with programmatic MLOp workflows, can lead to substantial performance gains. The combination of human-in-the-loop oversight and automated optimization (as shown in Tables2^3^ and Multimedia Appendix 1) offers a pragmatic framework for building biologically grounded classifiers in data-limited settings.

Few studies have systematically addressed classifier development requirements in very small cohorts. Our previous work demonstrated feasibility in a prospective cohort [6] without comparative evaluations against conventional methods or MLOp integration. Transfer learning has shown promise in classifying cell types in single-cell RNA sequencing [31] and transcriptomic datasets derived from large human cohorts [32], but these methods have not yet been applied specifically to small human cohorts for clinical predictive analytics.

Several limitations must be noted: (i) alternative machine learning models (SVM, Logistic Regression, XGBoost) consistently underperformed relative to RF, and results were omitted for brevity. Future research should explore fusion deep learning and transfer learning approaches. (ii) Our conclusions are based on limited datasets, necessitating additional transcriptomic data or simulation studies to robustly assess generalizability. (iii) Despite efforts to control overfitting, inherent constraints persist due to small sample sizes, emphasizing the need to develop microcohorts through subsampling larger paired-sample datasets in future studies; though such datasets are uncommon.

### Conclusions

Most of the approximately 65,000 known human diseases remain inadequately treated due to their rarity and the consequent scarcity of comprehensive studies. The low prevalence of these diseases severely limits conventional transcriptomic approaches as bulk RNA sequencing (bRNAseq) typically requires larger cohorts for effective classifier development. Emerging technologies such as spatial RNA sequencing and single-cell RNA sequencing present promising alternatives suitable for smaller cohort studies; however, these methods currently incur approximately 20 times higher costs per sample and capture around 5 times fewer mRNA transcripts. As these technologies become more affordable and achieve improved transcriptomic coverage, novel analytical methodologies tailored for small cohorts are expected to evolve. Additionally, transfer learning techniques, already successfully applied to large-scale transcriptomic datasets, offer considerable potential for small-cohort classification. However, standardized frameworks for applying transfer learning specifically to paired-sample designs are not yet established, highlighting an important area for future research.

This study systematically evaluated multiple complementary approaches designed to enhance the statistical power of bulk mRNA-based classification within microcohorts. Our results demonstrate that the integration of these approaches improves precision and recall by approximately 12.5%‐14.5% compared to traditional single-sample methodologies. Specifically, we propose strategies that include the following: (i) leveraging paired comparisons of affected and control tissues within individual subjects, and (ii) using MLOp-guided analytical workflows combined with expert-in-the-loop oversight to ensure robustness, transparency, and reproducibility. This paired-sample methodology has been shown to improve classifier development in large cohorts [6,33-38], and here we show that, in very small cohorts, it also facilitated classifier development at both the individual mRNA level (via fold-change analysis) and the biologically interpretable knowledge-anchored pathway level (through N-of-1-pathway-based analyses leveraging Gene Ontology gene sets). Our results indicate that both paired-sample representations can outperform single-sample approaches, with fold-change or pathway-based features proving more effective depending on the underlying biological context. The adoption of MLOps practices optimized hyperparameter tuning and model deployment, as well as mitigated overtraining, while expert oversight ensured the biological validity of the results. Collectively, these strategies effectively address the challenges posed by high feature dimensionality and limited sample sizes, thereby laying the groundwork for advancing personalized therapeutic interventions in rare disease contexts.

Although this investigation primarily targeted a specific transcriptomic scale, optimal classifiers for clinical prediction are likely to incorporate comprehensive data across diverse biological scales (metabolome, genome, proteome, methylome, etc) jointly with real-world evidence and clinical dimensions. Future research endeavors should integrate transcriptomic data with multiomics approaches, medical imaging, and patient-centric outcomes to further enhance predictive accuracy and personalized medicine capabilities.

## Supplementary material

10.2196/80735 Multimedia Appendix 1 Comparative analysis of the ML model and rationale for choosing RF.

10.2196/80735 Multimedia Appendix 2 Learning curve and power analyses for generalizability in microcohort transcriptomics.

10.2196/80735 Multimedia Appendix 3 Traceability of Random Forest–selected features to biological pathways and interpretive cautions.

10.2196/80735 Multimedia Appendix 4 Cross-validation accuracies with CIs.

10.2196/80735 Multimedia Appendix 5 Transcript-level feature reduction controls to evaluate dimensionality effects.

## Abbreviations

AIME: Artificial Intelligence in Medicine
AMIA: American Medical Informatics Association
BC: Breast Cancer dataset
bRNAseq: Bulk RNA sequencing
CV: cross-validation
FC: fold change
GO: gene ontology
HRV: human rhinovirus
LLMs: large language models
MDI: mean decrease impurity
ML: Machine Learning
MLOps: Machine Learning Operations
mRNA: Messenger RNA
PBMCs: peripheral blood mononuclear cells
PIK3CA: phosphatidylinositol-4,5-bisphosphate 3-kinase catalytic subunit alpha
RF: Random Forest
RMA: Robust Multi-array Average (normalization method)
RNA-seq: RNA sequencing
SVM: support vector machine
TCGA: The Cancer Genome Atlas
TCGA-BRCA: TCGA Breast Invasive Carcinoma project code
TMM: trimmed mean of M values
TP53: tumor protein p53
W&B: Weights & Biases MLOps
XGBoost: eXtreme Gradient Boosting
