# Supplementary material for: Paired-Sample and Pathway-Anchored MLOps Framework for Robust Transcriptomic Machine Learning in Small Cohorts: Model Classification Study
Source: JMIR Bioinform Biotechnol. 2025 Oct 8;6:e80735. doi: 10.2196/80735 (PMC12507327; doi:10.2196/80735)
Supplement: Multimedia Appendix 1 [file bioinform-v6-e80735-s001.pdf]

Supplement File 1**: Comparative analysis of the ML model and rationale for choosing RF**

We selected Random Forest (RF) for $p \gg n$ gene-expression modeling, as it has proven robust in high-dimensional transcriptomics(Breiman, 2001). Its bootstrap aggregation and feature subsampling reduce overfitting, handle multicollinearity, and capture nonlinear interactions with minimal tuning (Breiman, 2001). As shown in **Table S1**, across both cohorts, RF achieved the highest accuracy and lowest variance under cross-validation, outperforming SVM, logistic regression, and XGBoost. RF also yields feature-importance scores that we mapped to pathways for biological interpretability(Strobl et al., 2008).

Table S1: Comparative analysis of the ML models performance.

Bolded are the highest accuracies for each method in a dataset according to one feature (highest accuracy per horizontal line)**.** Legend: Baseline (non-MLOps) model comparison (Stratified 5-fold CV accuracy, mean). Identical preprocessing/feature spaces; fixed grids; no agents; lower than paper's MLOps results by design.

**Cohort**	**Feature representation**	**Overall Accuracies (without MLOps)**		
		**RF**	**XGBoost**	**SVM**
HRV	Single-sample mRNAs	**0.71**	0.68	0.65
HRV	Paired $\log_2$ Fold-Change	**0.78**	0.76	0.73
HRV	N-of-1 Pathways	**0.75**	0.70	0.67
Breast Cancer	Single-sample mRNAs	**0.70**	0.57	0.54
Breast Cancer	Paired $\log_2$ Fold-Change	**0.53**	0.48	0.45
Breast Cancer	N-of-1 Pathways	**0.62**	0.57	0.54

Supplement 1 References

Breiman L. *Random forests*. *Machine Learning*. 2001;45(1):5–32. doi:10.1023/A:1010933404324.

Strobl C, Boulesteix AL, Kneib T, Augustin T, Zeileis A. Conditional variable importance for random forests. *BMC Bioinformatics*. 2008;9:307. doi:10.1186/1471-2105-9-307.
