# Supplementary material for: Paired-Sample and Pathway-Anchored MLOps Framework for Robust Transcriptomic Machine Learning in Small Cohorts: Model Classification Study
Source: JMIR Bioinform Biotechnol. 2025 Oct 8;6:e80735. doi: 10.2196/80735 (PMC12507327; doi:10.2196/80735)

Supplement File 2. Learning-curve and power analyses for generalizability in micro-cohort transcriptomics. All experiments were executed end-to-end within a reproducible MLOps workflow. Configuration files (YAMLs), code commits, dataset and hyperparameter hashes, environment lockfiles, and figure/table scripts were versioned as immutable artifacts. Cross-validation folds were deterministically defined and logged, with the held-out test partition accessed only after model selection to prevent leakage. Hyperparameters were optimized through automated random-search sweeps over a pre-specified space (e.g., RF max_depth, max_features, min_samples_leaf, n_estimators). Each trial completed a full 5-fold CV, with the mean validation score across folds serving as the pre-defined selection objective. All per-trial and aggregate metrics, including fold identities and final configurations, were logged to Weights & Biases. This automation minimized researcher degrees of freedom, prevented data/code drift, and ensured hyperparameters were chosen strictly from out-of-sample evidence, an essential safeguard against overfitting in micro-cohort transcriptomics. Exemplar learning curves are shown in **Figures A** and **B.**

Figure A. N-of-1-pathways HRV 19 subjects. Top panel: 5-fold cross validation accuracy; LowerPanel: 5-fold cross validation precision.

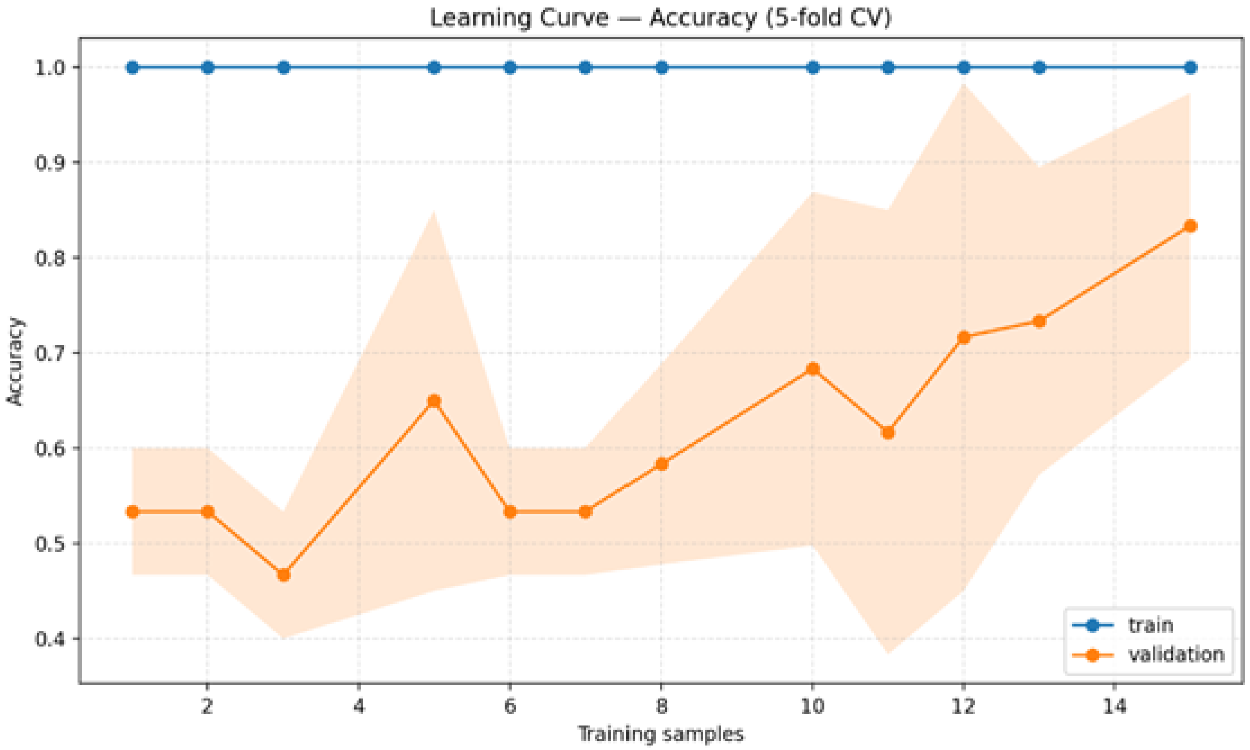

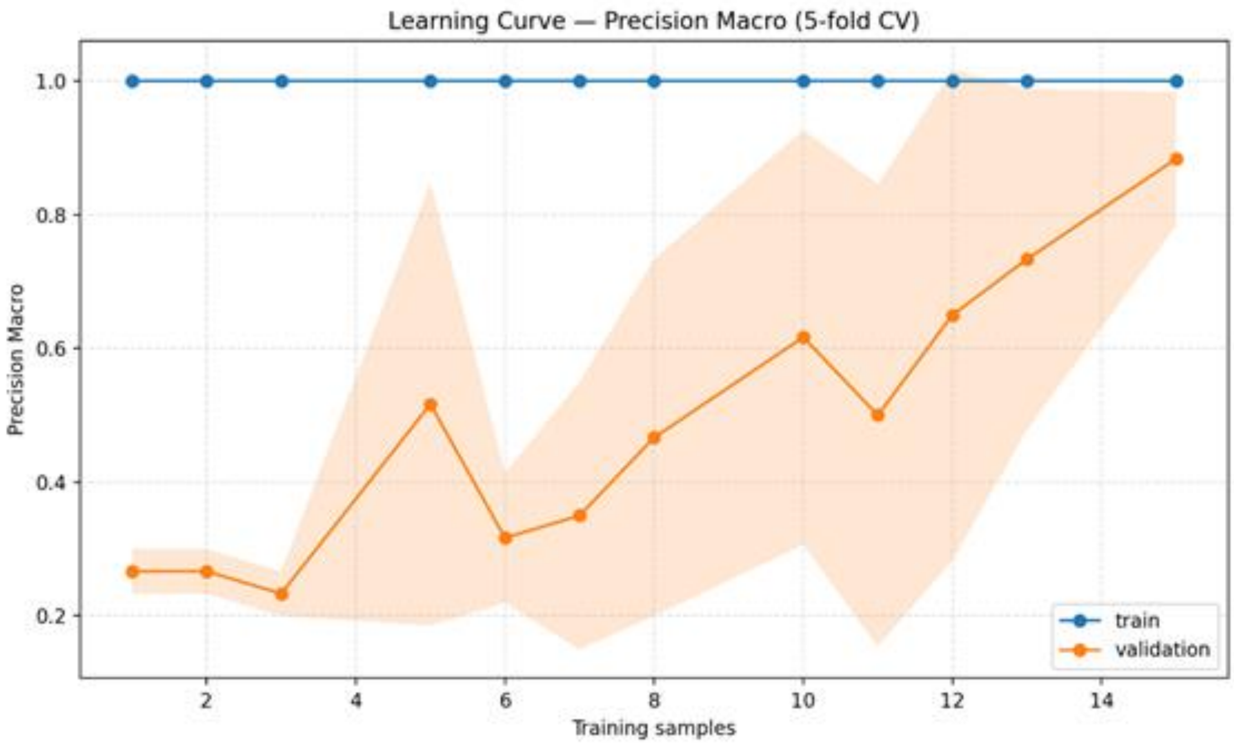

Figure B. N-of-1-Pathways BC 42 subjects as example increase. Top panel: 5-fold cross validation accuracy; Lower Panel: 5-fold cross validation precision.

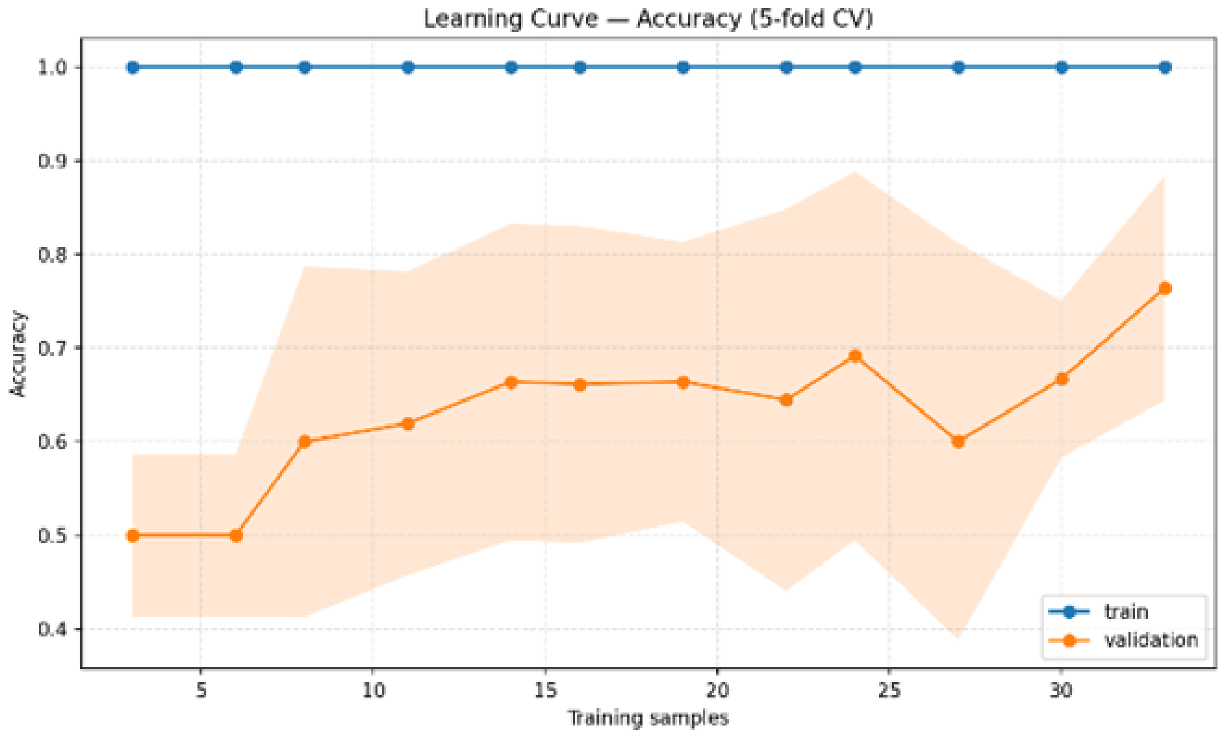

Supplement: Multimedia Appendix 2 [file bioinform-v6-e80735-s002.pdf]
