# Supplementary material for: Paired-Sample and Pathway-Anchored MLOps Framework for Robust Transcriptomic Machine Learning in Small Cohorts: Model Classification Study
Source: JMIR Bioinform Biotechnol. 2025 Oct 8;6:e80735. doi: 10.2196/80735 (PMC12507327; doi:10.2196/80735)

Supplementary File 3. Traceability of Random Forest–selected features to biological pathways and interpretive cautions

We conducted two complementary analyses to assess the biological interpretability of Random Forest–selected features: (i) heatmaps of prioritized features under unsupervised clustering and (ii) enrichment analysis of gene-set overlap. *For heatmaps,* we first present the two-sample N-of-1 pathway features, which yield a smaller and more interpretable set, followed by single-sample RF classifiers and two-sample fold-change representations for comparison. Heatmaps were generated in R using the *pheatmap* package with Ward.D2 clustering on Euclidean distances (HRV) or Manhattan distances (BC). *For enrichment,* we used curated Gene Ontology Biological Process (GOBP) sets (Ashburner et al., 2000), filtered to include 15–500 genes overlapping with the assay universes: 20,501 genes from the breast cancer RNA-seq platform and 20,106 genes from the HRV microarray platform. This yielded 4,815 GOBPs for BC and 4,813 GOBPs for HRV. We then tested the significance of overlap between prioritized genes and GOBPs using Fisher's exact test, with the platform-specific gene universes as background. Multiple testing was corrected using the Benjamini–Hochberg method (Benjamini et al., 1995) implemented in R.

As shown in **Table S3.1**, all four enrichment analyses of classifier-selected transcripts (STRING protein interactions and GO Biological Processes) failed to yield results at a false discovery rate <5%. This is consistent with the idea that classifiers preferentially select uncorrelated features that improve discrimination rather than mechanistic coherence. Similarly, only a few GO Biological Processes prioritized by the N-of-1 pathway–based RF classifiers showed hierarchical relationships. These findings underscore a central caution: features identified by predictive models should not be ascribed mechanistic or biological meaning without independent evidence from in vivo studies or group comparison analyses. Classifier-selected features are optimized for class separation, not causal inference, and many alternative gene sets can achieve comparable accuracy because of the correlation structure of transcriptomic data. Such features are often unstable and dataset-dependent, with small perturbations producing different sets of predictors even when performance remains unchanged. As emphasized by Simon and colleagues, the primary value of a classifier lies in its predictive performance and rigorous validation, not in mechanistic interpretation of individual features (Simon et al., 2003; Simon, 2005).

Tabel S3.1. Enrichment Analyses of Transcripts and Associated Heatmaps. * Note: The HRV cohort input consisted of 20,502 RMA-normalized Affymetrix GeneChip probe-set expressions. Of these, 266 prioritized probe-sets mapped to 240 unique genes

.Cohort	RF analysis- design (See Method)	Count of prioritized features by RF	Count of the inferred GOBPs at Fisher's exact test FDR corrected p-value <0.05
HRV	Single mRNA	240 (266 probe-set) *	0
	Fold change	112	0
BC	Single mRNA	105	0
	Fold change	97	0

References:

Simon R, Radmacher MD, Dobbin K, McShane LM. Pitfalls in the use of DNA microarray data for diagnostic and prognostic classification. Journal of the National Cancer Institute. 2003;95(1):14–18. doi:10.1093/jnci/95.1.14.

Simon R. Roadmap for developing and validating therapeutically relevant genomic classifiers. Journal of Clinical Oncology. 2005;23(29):7332–7341. doi:10.1200/JCO.2005.02.8712.

Ashburner, M., C. A. Ball, J. A. Blake, D. Botstein, H. Butler, J. M. Cherry, A. P. Davis, et al. 2000. "Gene Ontology: Tool for the Unification of Biology. The Gene Ontology Consortium." Nature Genetics 25 (1): 25–29. https://doi.org/10.1038/75556.

Benjamini Y, Hochberg Y. Controlling the False Discovery Rate: A Practical and Powerful Approach to Multiple Testing. Vol. 57, Source: Journal of the Royal Statistical Society. Series B (Methodological). 1995.

Figure S3.1. Heatmaps of Two-Sample N-of-1 pathway scores for Random Forest–selected features in HRV and breast cancer. Ternary N-of-1 pathway scores are shown (–1 = significantly downregulated, 0 = not significant, 1 = significantly upregulated) when comparing paired samples (infection vs. baseline in HRV; TP53 vs. PIK3CA mutations in BC). These analyses illustrate how prioritized features map onto biological processes, supporting interpretability while warranting cautious inference.
Left Panel (HRV). In symptomatic HRV infections, interferon response pathways (GO:0034340) were upregulated in 8/9 subjects (W+), but mostly unchanged or downregulated in asymptomatic cases. Conversely, T-cell receptor signaling (GO:0050852) was downregulated in 6/9 symptomatic subjects (W–) and generally unchanged or upregulated in asymptomatic cases, reflecting contrasting innate versus adaptive immune activity.
Right Panel (Breast Cancer). Among oncogene-driven tumors, >90% of TP53-mutated cases versus ~45% of PIK3CA-mutated cases showed reduced activity in cell cycle–related processes (GO:0000083 G1/S transition regulation, GO:0034508 centromere assembly, GO:1902850 mitotic microtubule organization). This aligns with TP53's role as a tumor suppressor and highlights distinct downstream biology between TP53- and PIK3CA-driven tumors.

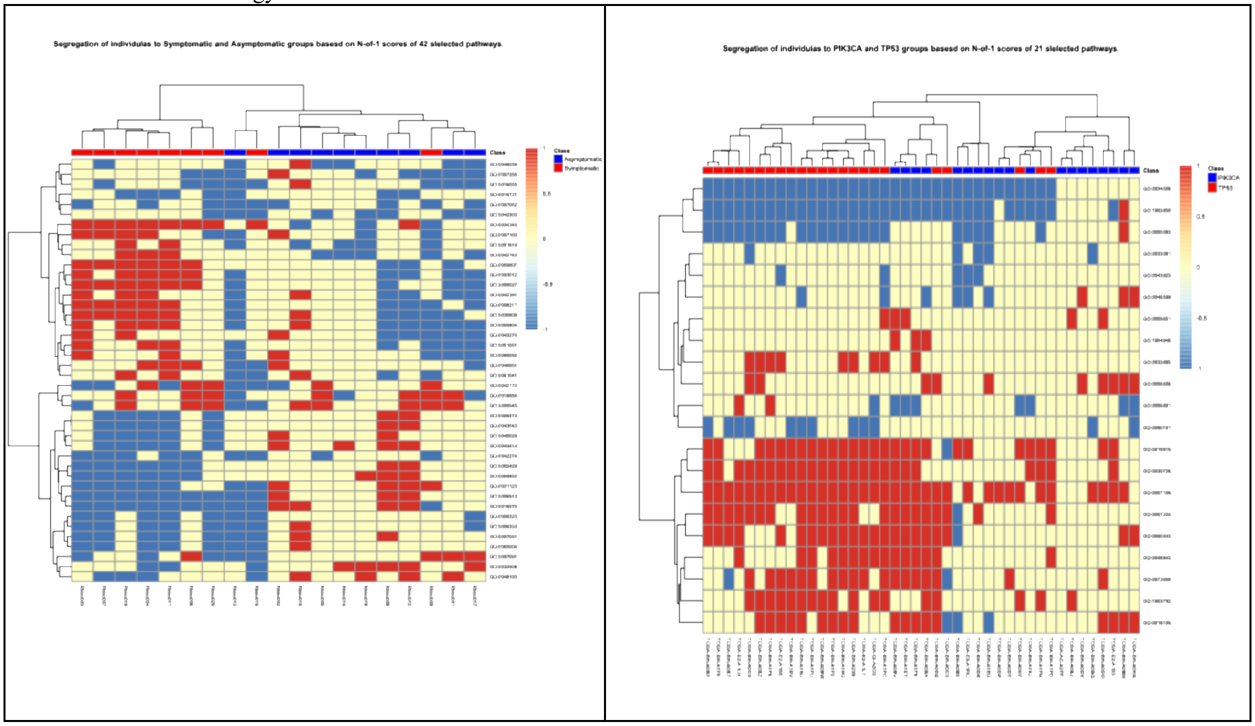

Figure S3.2 Heatmap of log_2 expression values for 240 RF-prioritized genes in the HRV cohort using single-sample classifiers

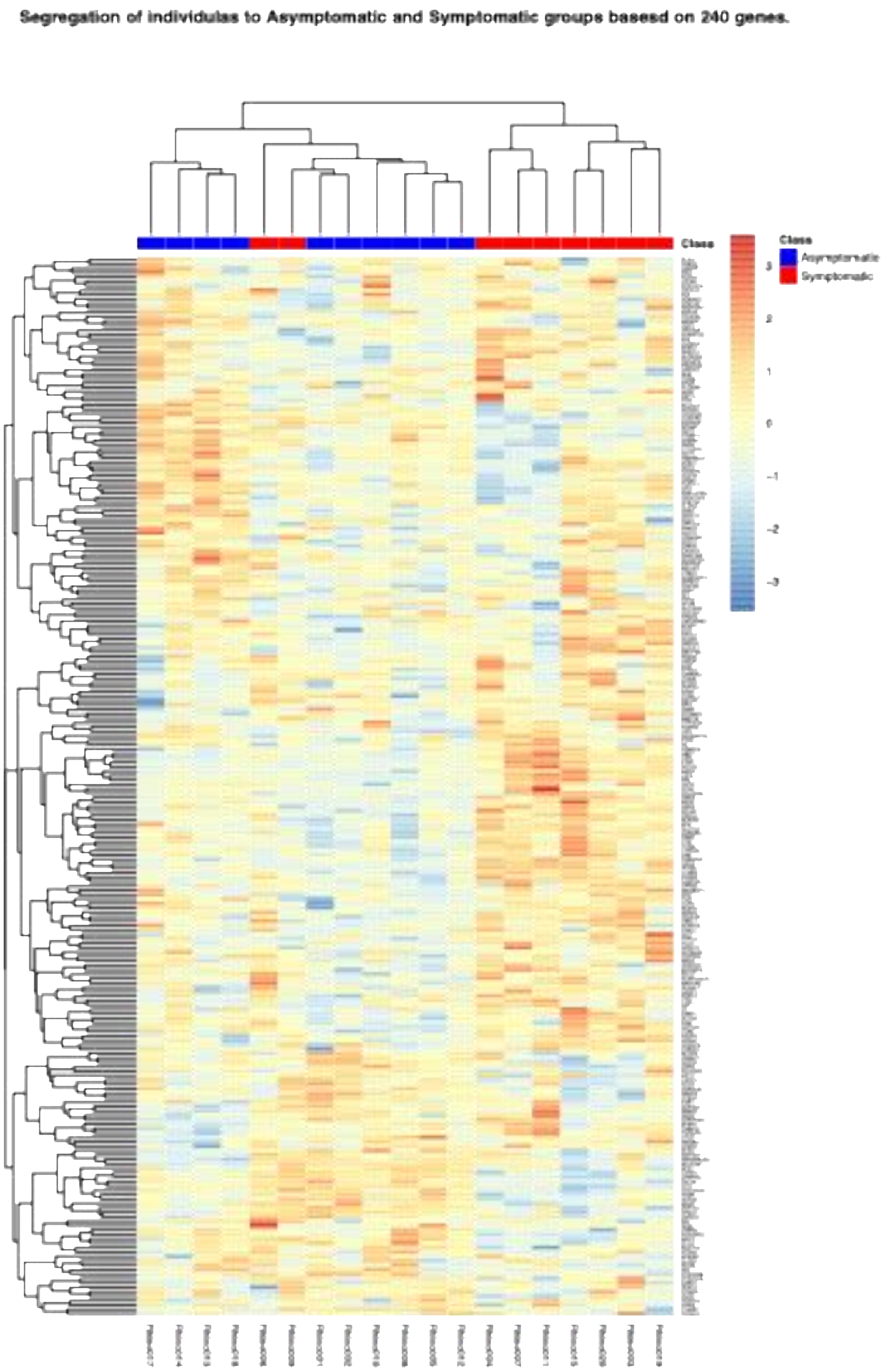

Suppl Fig 3.3. Heatmap of log_2 expression values for 112 RF-prioritized genes in the HRV cohort using two-sample FC classifiers

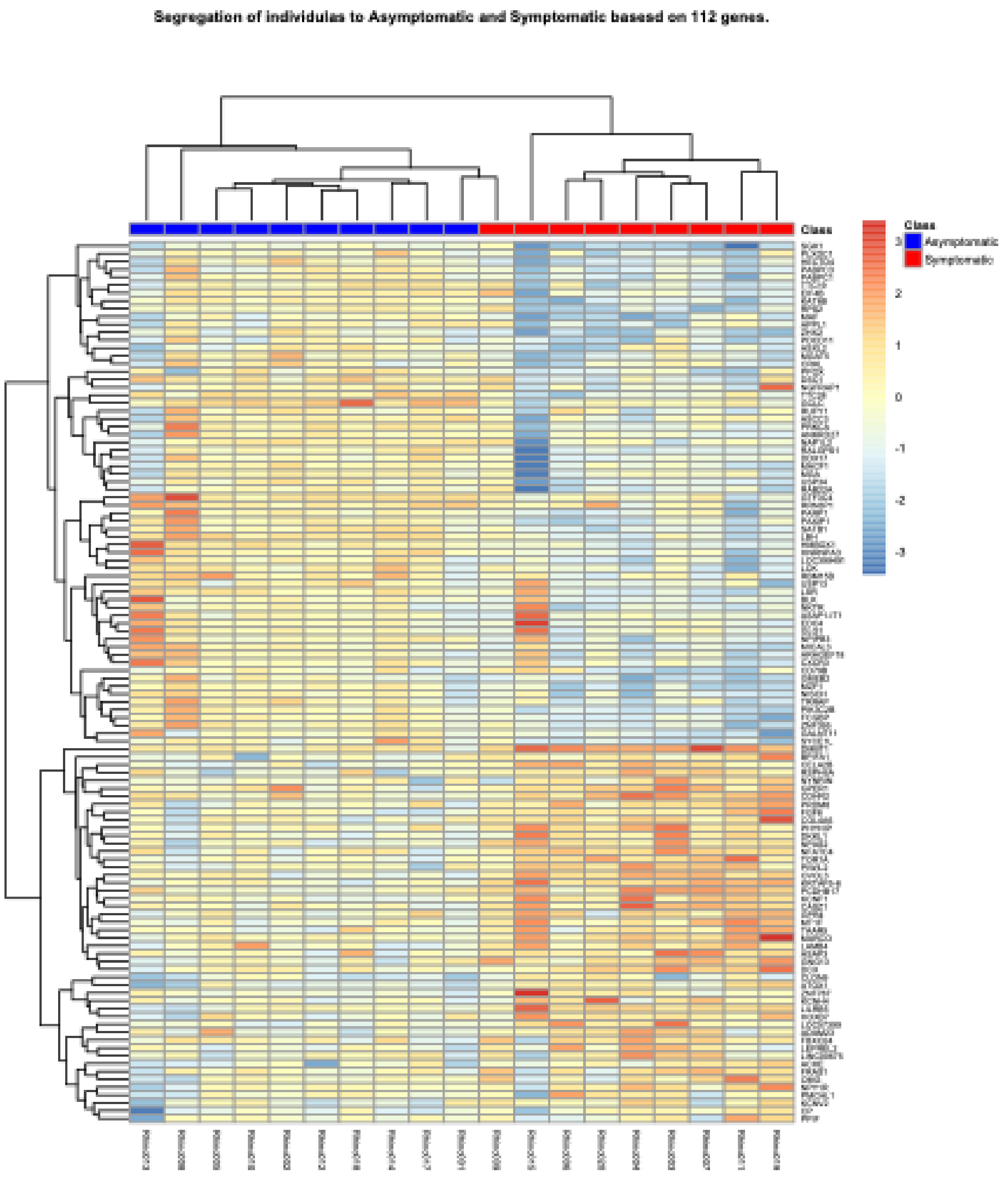

Suppl Fig 4. Heatmap of log_2 expression values for 105 RF-prioritized genes in the BC cohort using single-sample classifier

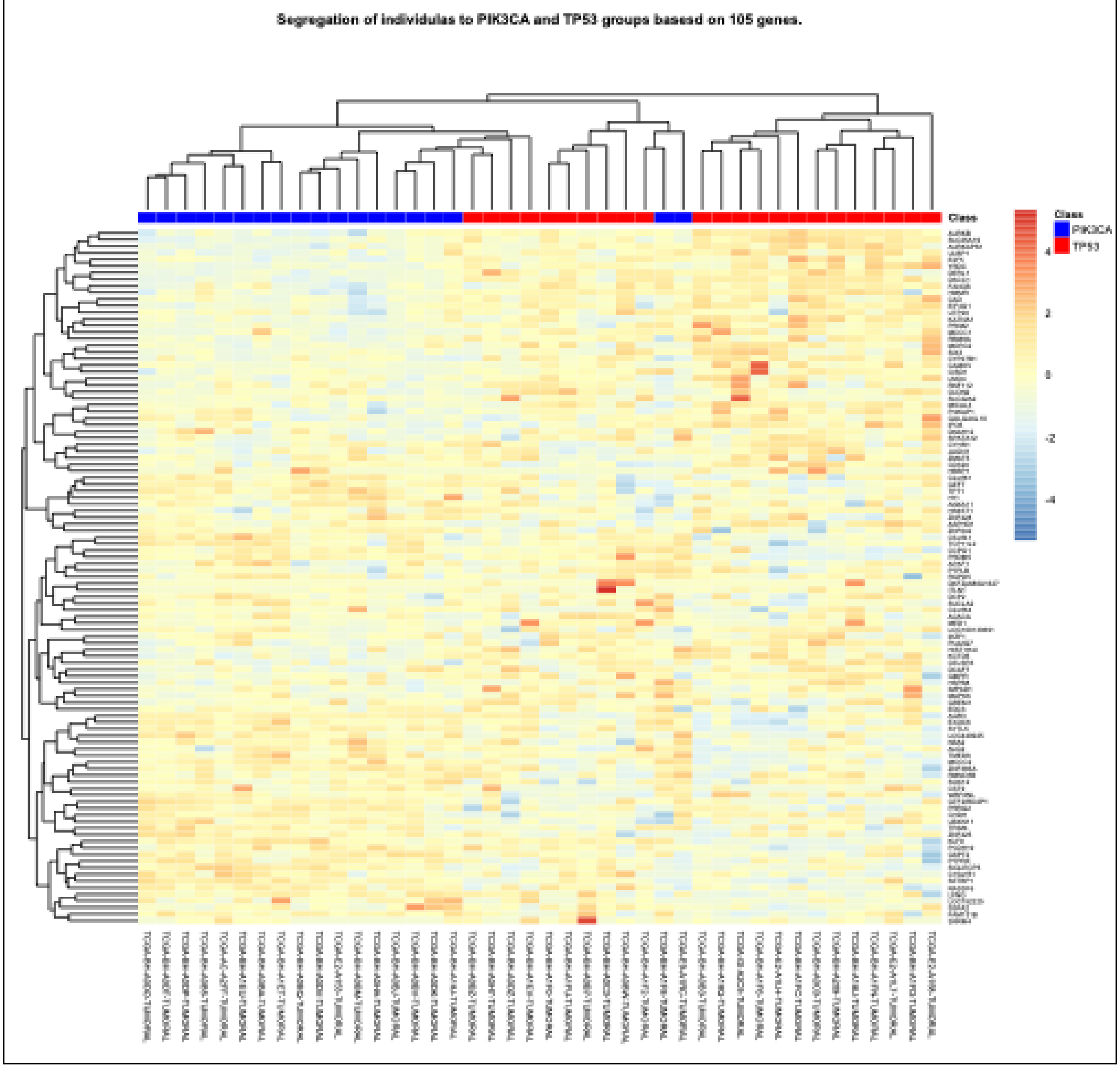

Suppl Fig 5. Heatmap of log_2 expression values for 97 RF-prioritized genes in the HRV cohort using two-samples FC classifiers

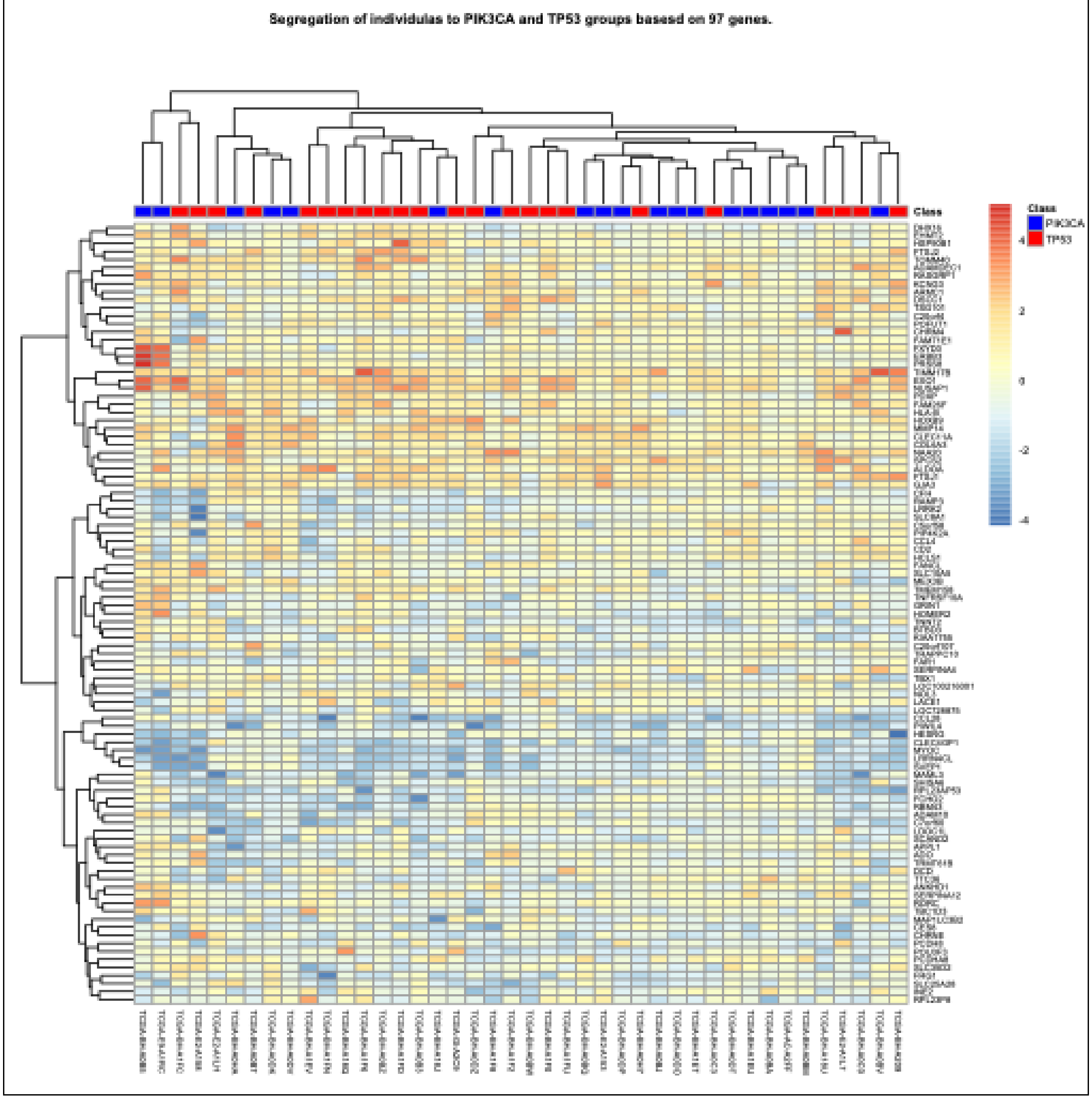

Supplement: Multimedia Appendix 3 [file bioinform-v6-e80735-s003.pdf]
