# Supplementary material for: Paired-Sample and Pathway-Anchored MLOps Framework for Robust Transcriptomic Machine Learning in Small Cohorts: Model Classification Study
Source: JMIR Bioinform Biotechnol. 2025 Oct 8;6:e80735. doi: 10.2196/80735 (PMC12507327; doi:10.2196/80735)
Supplement: Multimedia Appendix 4 [file bioinform-v6-e80735-s004.pdf]

Supplement File 5 . Cross-validation accuracies with confidence intervals. Confidence intervals were approximated from the mean and standard deviation of accuracies obtained across folds, as original fold-level distributions were not stored.

Table S5.1. Performance summary of analysis in HRV RF classifier with Confidence Intervals

	Two sample (one in each condition) mRNAs	
**Single sample mRNAs**	**Fold Change**	**Nof1-Pathways**
CV Accuracy: .85 ± .16	CV Accuracy: .95 ± .15	CV Accuracy: .88 ± .14
90% CI (.70-1)	90% CI (.81-1)	90% CI (.75-1)

Table S5.2. Performance summary analysis in BC RF classifier with Confidence Intervals

	Two sample (one in each condition) mRNAs	
**Single mRNAs**	**Fold Change**	**Nof1-Pathways**
CV Accuracy: .72 ± .18	CV Accuracy: .62 ± .15	CV Accuracy: **.73 ± .07**
90% CI (.55-89)	90% CI (.48-.76)	90% CI (.66-.80)
