# Supplementary material for: Paired-Sample and Pathway-Anchored MLOps Framework for Robust Transcriptomic Machine Learning in Small Cohorts: Model Classification Study
Source: JMIR Bioinform Biotechnol. 2025 Oct 8;6:e80735. doi: 10.2196/80735 (PMC12507327; doi:10.2196/80735)
Supplement: Multimedia Appendix 5 [file bioinform-v6-e80735-s005.pdf]

Supplement File 4. Transcript-level feature reduction controls to evaluate dimensionality effects

Table S4.1 HRV-Random Forest Cross-Validation Summary for HRV with 533 mRNAs. Values are mean ± SD across cross-validation folds. Hyperparameters are condensed for compact reporting.

**Run A — RF Single transcripts 553 mRNAs**	**Run B — RF Fold change 553 mRNAs**
CV Accuracy: .90 ± .20<br>**CV Precision: .85 ± .30**<br>CV Recall: .90 ± .20	CV Accuracy: .95 ± .10<br>**CV Precision: .97 ± .07**<br>CV Recall: .95 ± .10
Hyperparameters: criterion= entropy; max_depth=113; max_features= log2; n_estimators=256; random_seed=42	Hyperparameters: criterion=entropy; max_depth=134; max_features= sqrt; n_estimators=59;

Table S4.2 BC-Random Forest Cross-Validation Summary for HRV with 4442 mRNAs (Two Runs). Values are mean ± SD across cross-validation folds**.** Hyperparameters are condensed for compact reporting.

**Run A — RF Single transcripts 4442 mRNAs**	**Run B — RF Fold change 4442 mRNAs**
CV Accuracy: .87 **± .17**<br>CV Precision: **.84 ± .21**<br>CV Recall: .80 **± .22**	CV Accuracy: .78 **± .11**<br>CV Precision: **.85 ± .06**<br>CV Recall: .75 **± .1**
Hyperparameters: criterion=gini; max_depth=9; max_features=sqrt; n_estimators=34; random_seed=42	Hyperparameters: criterion=entropy; max_depth=40; max_features=log2; n_estimators=43; random_seed=42
